# The transcriptional regulator TBX3 promotes progression from non-invasive to invasive breast cancer

**DOI:** 10.1186/s12885-016-2697-z

**Published:** 2016-08-23

**Authors:** Milica Krstic, Connor D. Macmillan, Hon S. Leong, Allen G. Clifford, Lesley H. Souter, David W. Dales, Carl O. Postenka, Ann F. Chambers, Alan B. Tuck

**Affiliations:** 1 Department of Oncology, Schulich School of Medicine and Dentistry, Western University, London, ON Canada; 2 The Pamela Greenaway-Kohlmeier Translational Breast Cancer Research Unit, London Regional Cancer Program, London Health Sciences Centre, London, ON Canada; 3 Department of Pathology, Schulich School of Medicine and Dentistry, Western University, London, ON Canada; 4 Department of Surgery, Schulich School of Medicine and Dentistry, Western University, London, ON Canada

**Keywords:** Breast cancer, Ductal carcinoma in situ, Epithelial-mesenchymal transition, Invasive mammary carcinoma, TBX3

## Abstract

**Background:**

TBX3 is a T-box transcription factor repressor that is elevated in metastatic breast cancer and is believed to promote malignancy of tumor cells, possibly by promoting cell survival and epithelial-mesenchymal transition.

**Methods:**

The relative expression of TBX3 was assessed in the 21T cell lines which were derived from an individual patient and represent three distinct stages of breast cancer progression: 21PT, atypical ductal hyperplasia; 21NT, ductal carcinoma in situ; and 21MT-1, invasive mammary carcinoma. Two different isoforms of TBX3 (TBX3iso1 and TBX3iso2) were overexpressed to evaluate cell survival/colony forming ability, growth, and invasion in the ductal carcinoma in situ-like 21NT cell line using an in vitro Matrigel model of cancer progression. In addition, TBX3 expression was knocked down to evaluate the effects of downregulating TBX3 on the invasive mammary carcinoma-like 21MT-1 cell line. Finally, PCR array profiling was used to assess alterations in gene expression due to TBX3 overexpression in the 21NT cells.

**Results:**

TBX3 is abundant in the invasive 21MT-1 cell line, while being minimally expressed in the non-invasive 21NT and 21PT cell lines. Overexpression of either TBX3iso1 or TBX3iso2 in 21NT cells resulted in increased cell survival/colony forming ability, growth vs. apoptosis and invasion in Matrigel. In contrast, short hairpin RNA-mediated knockdown of TBX3 in the 21MT-1 cells resulted in smaller colonies, with a more regular, less dispersed (less infiltrative) morphology. Array profiling of the 21NT TBX3 iso1 and iso2 transfectants showed that there are common alterations in expression of several genes involved in signal transduction, cell cycle control/cell survival, epithelial-mesenchymal transition and invasiveness.

**Conclusions:**

Overall, these results indicate that TBX3 (isoform 1 or 2) expression can promote progression in a model of early breast cancer by altering cell properties involved in cell survival/colony formation and invasiveness, as well as key regulatory and EMT/invasiveness-related gene expressions.

## Background

Arguably the most critical stage of early breast cancer progression is the transition from in situ (ductal carcinoma in situ, DCIS) to invasive (invasive mammary carcinoma, IMC) disease. Although a number of molecular changes have been identified that accompany invasive breast cancer [1–6], those that can directly control the transition from DCIS to IMC remain elusive.

Using microarray analysis, we previously identified T-box transcription factor 3 (TBX3) as a potential regulator of progression from DCIS to IMC, using the 21T cell lines which represent distinct stages of breast cancer progression [7]. Specifically, we found that invasive, metastatic 21MT-1 cells expressed higher levels of TBX3 than non-invasive, DCIS-like 21NT cells or non-invasive, atypical ductal hyperplasia (ADH)-like 21PT cells [7]. TBX3 is a member of the T-box family of transcription factors that play an important role in development of many animal species. In mouse embryo development, a model has emerged in which TBX3 expression is both induced and maintained in early mammary gland initiation by Wnt and fibroblast growth factor (FGF) [8]. In humans, Ulnar-mammary syndrome, a congenital autosomal dominant disorder, is caused by mutations that result in haploinsufficiency of TBX3 and is characterized by upper-limb anomalies and mammary gland hypoplasia [9].

TBX3 has been linked to tumorigenesis and is involved in cell cycle control and inhibition of cell senescence, through both p53-dependent and independent pathways [10, 11]. The p53-dependent pathway signals through p14^ARF^, a tumor suppressor and cell cycle control protein that is a product of the cyclin-dependent kinase inhibitor 2A (CDKN2A) gene, along with p16^INK4A^. TBX3 directly represses transcription of p14^ARF^ [10, 12]. Downregulation or inhibition of p14^ARF^ leads to increased proliferation and immortalization, as well as failure of apoptosis [12]. Aside from its role in the cell cycle, TBX3 is a known repressor of E-cadherin expression in melanoma, leading to enhanced invasiveness [13, 14]. TBX3 expression has also been found to be associated with cell survival in hepatocellular carcinoma, where it is induced by Wnt/β-catenin signalling [15].

Two different isoforms of TBX3 have been identified, TBX3iso1 and TBX3iso2. The TBX3iso2 variant has an extra 20 amino acids, encoded by exon 2a, inserted into the T-box domain [9]. As the 2a insertion is within the T-box domain, which is required for DNA-binding and protein-protein interactions, it was initially proposed that this variant may have altered DNA-binding properties, and that it may in fact interfere with the senescence-inhibiting properties of the other isoform [16]. However, it has been found that TBX3iso2 (also referred to as TBX3 + 2a because of the presence of exon 2a) can indeed bind the DNA-binding site and act as an anti-senescence factor [17].

Here we examined whether either or both isoforms of TBX3 could influence breast cancer progression, in particular the transition from non-invasive to invasive disease. We show that both isoforms of TBX3 have a similar functional effect in promoting breast cancer progression to a more malignant phenotype, and identify TBX3-induced changes in expression of genes involved in signal transduction, cell cycle control/cell survival and epithelial-mesenchymal transition (EMT)/invasiveness that may play a role in this progression.

## Methods

### Cell lines and culture conditions

The 21T parental cell lines (21PT, 21NT, and 21MT-1) were a kind gift from Dr. Vimla Band (Dana Farber Cancer Institute, Boston, MA) [18] and were cultured in alpha modification of Eagle’s medium supplemented with 2.8 μM hydrocortisone (H), 12.5 ng/ml epidermal growth factor (E), 2 mM L-glutamine, 1 μg/ml insulin, 10 mM HEPES, 1 mM sodium pyruvate, 0.1 mM non-essential amino acids and 50 μg/ml gentamycin reagent (called αHE – all supplements from Wisent Bioproducts). For regular culture conditions, the αHE media was further supplemented with 10 % fetal bovine serum (FBS) (Sigma-Aldrich) and named αHE10F. Stably transfected cells were cultured in αHE10F containing either 500 μg/ml G418 (Wisent Bioproducts) or 0.8 μg/ml puromycin (Sigma-Aldrich).

### Generation of TBX3 expression vectors

Expression vectors were constructed for each TBX3 isoform (TBX3iso1and TBX3iso2). To obtain TBX3iso2, PCR amplification of the whole transcript was performed from a pOTB7 expression vector containing TBX3iso2 [Genbank:BC025258] (Open Biosystems Thermo Scientific) as the cDNA template using Phusion High-Fidelity DNA Taq polymerase (New England BioLabs). Primers used to amplify TBX3iso2 were: forward: 5′-GCC ACC ATG AGC CTC TCC ATG AGA-3′ and reverse: 5′-TTC GGG ACC GCC TGC GGG ACC TGT CCG GC-3′. To produce TBX3iso1, two PCR product fragments, representing the transcript before (fragment 1) and after (fragment 2) the 66 bp TBX3iso2 addition, were PCR amplified. Primers used for fragment 1 of TBX3iso1 were: forward: 5′-GCC ACC ATG AGC CTC TCC ATG AGA-3′ and reverse: 5′-CAT GGA GTT CAA TAT AGT AAA TCC ATG TTT GAC-3′. Primers used for fragment 2 of TBX3iso1 were: forward: 5′-TGG ATT TAC TAT ATT GAA CTC CAT GCA CAA AT-3′ and reverse: 5′-TTC GGG ACC GCC TGC GGG ACC TGT CCG GC-3′. All primers used for generation of the TBX3 expression vectors were purchased from Sigma-Aldrich. Products were separated on 1 % agarose gel and bands representing TBX3iso2 at ~2000 kb were extracted and pooled. For TBX3iso1, a band at ~660 kb for fragment 1 and a band at ~1380 kb for fragment 2 were gel extracted and pooled. When designing the PCR primers for the two fragments of TBX3iso1, the reverse primer of the upstream amplicon (fragment 1) and the forward primer for the downstream amplicon (fragment 2) had a 20 bp overlap to ensure that the ends of these amplicons would anneal in a subsequent PCR reaction to melt the fragments together. This annealed TBX3iso1 PCR product was purified and amplified again. After running this PCR product on a 1 % agarose gel and extracting at ~2000 bp, both TBX3iso1 and TBX3iso2 PCR products were incubated with T4 polynucleotide kinase (New England BioLabs), and purified. Both products were inserted separately into pZsGreen-C1 plasmids (Clonetech Laboratories), such that the ZsGreen was fused to the C-terminus of TBX3. To prepare the pZsGreen-C1 plasmid, it was digested with Afe1 (New England BioLabs) and incubated with calf intestinal alkaline phosphatase (New England BioLabs) to dephosphorylate the cut ends. The digested plasmid was electrophoresed and gel extracted. The plasmid and TBX3 PCR products were incubated overnight with ATP and T4 DNA Ligase (New England BioLabs) at 16 °C to complete ligation. Competent bacteria (DH5alpha) were transformed with the ligated plasmid and kanamycin resistant clones were expanded to isolate DNA. Clones were digested with the following enzyme pairs to check for proper size and orientation: AgeI/KpnI, MfeI/XhoI, and NheI/HndIII. Suitable clones were sequenced (DNA Sequencing Facility at Robarts Research Institute, London, ON).

The use of untagged TBX3 was required so the TBX3 expression vectors underwent site directed mutagenesis in order to introduce a stop codon at the end of full length TBX3iso1 and TBX3iso2 using QuikChange Site-Directed Mutagenesis Kit (Agilent Technologies) according to the manufacturer’s protocol. Clones were sent to the DNA Sequencing Facility at Robarts Research Institute for sequencing. Clones with the proper sequence were used for stable transfections.

### Transfections

Purified TBX3iso1 and TBX3iso2 plasmid DNA was transfected using PolyJet DNA In Vitro Transfection Reagent (SignaGen Laboratories) following the manufacturer’s protocol. Empty vector (EV) plasmids were transfected as a control. Stable transfectants were selected in αHE10F containing 500 μg/ml G418 (Wisent Bioproducts). Approximately two weeks post-transfection, resistant clones were pooled, expanded, and frozen for later use.

### Generation of lentiviral particles and transduction

Generation of short hairpin (sh) RNA containing lentivirus particles and knockdown of target genes were described previously [19, 20]. The shRNA target sequence for TBX3 was GCA TAC CAG AAT GAT AAG ATA which targeted the coding sequence of both TBX3iso1 and TBX3iso2. The shRNA target sequence for Luciferase (off-target knockdown control) was ACG CTG AGT ACT TCG AAA TGT. The TBX3 shRNA lentivirus particles generated were used to knockdown TBX3 in the 21MT-1 cell line and Luciferase shRNA was used a negative control. One week post-transduction, stable clones were selected in αHE10F containing 0.8 μg/ml puromycin (Sigma-Aldrich) and resistant clones were pooled, expanded, and frozen for later use.

### Isolation of RNA and quantitative real-time polymerase chain reaction (qRT-PCR)

Cells were harvested with trypsin and RNA was isolated using the RNeasy Mini Kit (Qiagen). Samples were treated with 30U DNase I (Qiagen), and 500 ng of RNA was converted into cDNA with the Superscript III First-Strand Synthesis System (Invitrogen) using Oligo (dT) 12–18 primers (Invitrogen). qRT-PCR was performed using RT2 SYBR Green ROX qPCR Mastermix (Qiagen,) on an Mx3000P QPCR system. Primers used for total TBX3 were: forward: 5′-CGC TGT GAC TGC ATA CCA GA-3′ and reverse: 5′-GTG TCC CGG AAA CCT TTT GC-3′. Primers used for TBX3iso1 were: forward: 5′-AGT GGA TGT CCA AAG TCG TCA C-3′ and reverse: 5′-CAT GGA GTT CAA TAT AGT AAA TCC ATG TTT GTC TG-3′. Primers used for TBX3iso2 were: forward: 5′-AGT GGA TGT CCA AAG TCG TCA C-3′ and reverse: 5′-CAC TTG GGA AGG CCA AAG TAA ATC CAT G-3′. Primers used for GAPDH were: forward: 5′-AGG CTG GGG CTC ATT TGC AG-3′ and reverse: 5-‘CCA TCC ACA GTC TTC TGG GTG-3′. All primers were purchased from Sigma-Aldrich. Output values were reported relative to GAPDH as fold expression normalized to control cell lines.

### Preparation of 21T cell lysates

Radioimmunoprecipitation (RIPA) buffer (10nM Tris-HCl pH 7.5, 1 mM EDTA, 0.5 mM EGTA, 150nM NaCl, 1 % Triton-X 100, 0.5 % DOC, and 1 % SDS) containing one protease inhibitor tablet per 10 ml (Complete, Mini, EDTA-free Protease Inhibitor Cocktail, Roche) was used to lyse cells grown as subconfluent monolayers in 10 cm dishes. The cells were removed with a cell scraper, collected in a clean microcentrifuge tube, and placed on a rotator for 20 min at 4 °C. Tubes were spun at 13,000 RPM for 10 min at 4 °C and the resulting supernatant was collected.

### Electrophoresis and Western blotting

Proteins were electrophoresed on 10 % polyacrylamide gels and subsequently analyzed after transfer by Western blot with mouse anti-TBX3 antibody (Abcam, ab58264; 1:2,000), anti-Vimentin (Dako, clone 3B4; 1:1,000), anti-Twist (Abcam, ab50887; 1:1,000), anti-Src (Cell Signaling, 2108; 1:1,000), mouse anti-β-actin antibody (Abcam, ab49900; 1:150,000), and anti-Vinculin (Sigma, V9264; 1:40,000). After incubation with the appropriate horseradish peroxidase-conjugated secondary antibody (anti-mouse, Amersham GE Healthcare; anti-rabbit, Sigma) diluted 1:10,000, protein bands were detected using ECL Plus Western Blotting Detection Reagents (Amersham GE Healthcare) and then exposed to film in a dark room. Densitometric quantification was performed using ImageJ (Open source software, National Institutes of Health, USA).

### Immunohistochemistry of 21T cell pellets

Trypsinized cells were washed twice in phosphate buffered saline (PBS), and pelleted in 15 ml conical tubes. Pellets were re-suspended in 10 % neutral buffered formalin (NBF) and stored at 4 °C overnight. Formalin was removed, and pellets re-suspended in 1 ml of 1 % agarose cooled to 35 °C. The hardened pellets were wrapped in lens paper, cassetted, and processed to paraffin. For histology, 4 μm sections were deparaffinized, pre-treated with 10 mM citrate buffer pH = 6 and blocked for endogenous peroxidises in 3 % H_2_O_2_/methanol. Sections were immunostained with rabbit anti-TBX3 antibody (Abcam, ab99302) diluted 1:300 at 4 °C overnight. Signal detection was accomplished using ThermoScientific UltraVision LP Detection System (TL-060-HD).

### Three-dimensional Matrigel culture and immunofluorescence

Three-dimensional Matrigel culture was described previously [20–22]. Following a 9 day growth period, images were taken at both 4X and 10X objective using an inverted microscope. ImageJ (Open source software) was used to determine colony formation rates by quantifying the percentage of the population that formed colonies greater than 50 μm in diameter. ImageJ was also used to determine the proportion of circular colonies; a binary quantification method was utilized, with a circular colony having a circularity index above 0.75.

For immunofluorescence of Matrigel cultures, after 9 days of growth, the Matrigel plugs were fixed (10 % NBF) and permeabilized (0.5 % Triton X-100 in PBS). After blocking with normal goat serum (Invitrogen), rabbit anti-Ki67 (Abcam, ab833) and anti-cleaved caspase 3 (Cell Signaling, 9661) antibodies were incubated at 1:150 dilution in 10 % normal goat serum overnight at 4 °C. Cells were incubated with Alexa Fluor 488 Goat Anti-Rabbit secondary antibody (Invitrogen, A11034) and counterstained with Hoechst 33342 (Invitrogen, H1399) and Alexa Fluor 546 Phalloidin (Invitrogen, A22238). Coverslips were mounted over the stained Matrigel plugs with ProLong Gold Antifade Reagent (Invitrogen). Imaging was done using an Olympus Confocal Imaging System (FluoView FV1000 coupled to the IX81 Motorized Inverted System Microscope). Nuclei per colony were manually counted after acquiring 3D colony images.

### Transwell migration and invasion assays

To assess the migratory potential of 21NT cells overexpressing TBX3, transwell inserts with 8.0 μm pores (Corning, 3422; 24-well plate) were coated with a thin layer of gelatin (6 μg) to serve as a substrate for migration without obstructing movement through the pores. A 100 μl cell suspension (5 × 10^5^ cells/ml in αHE with 0.1 % bovine serum albumin (BSA)) was added to the upper chambers and 0.75 ml αHE10F media was added to the bottom wells. After incubation at 37 °C and 5 % CO_2_ for 22 h, migrated cells were fixed with 1 % gluteraldehyde for 20 min and stained with full-strength Hematoxylin for 15 min. Cells that remained on top of the membrane were removed using a cotton swab. Images of 5 non-overlapping fields of view were acquired using Image-Pro Analysis Software (Media Cybernetics) coupled to an inverted microscope at 10X objective. Cells were counted from the acquired images using ImageJ (Open source software). Similarly, to assess the invasive potential, transwell inserts with 8.0 μm pores precoated with Matrigel (Corning, 354480) were used and incubation lasted 22 h. Means derived from four replicates were used during analysis.

### RT^2^ PCR arrays

RNA was isolated from TBX3iso1 and TBX3iso2 transfectants, as well as vector controls, using the RNeasy Mini Kit (Qiagen). One microgram of RNA was converted into cDNA using the RT2 First Strand Kit (Qiagen). RT2 SYBR Green Mastermix was combined with the cDNA and dH_2_O as per the manufacturer’s protocol. Expression of 84 key genes commonly involved in dysregulation of signal transduction pathways and other biological processes involved in breast cancer were assessed using Human Breast Cancer RT^2^ PCR arrays (Qiagen, PAHS-131ZA-2). Data analysis was conducted by SA Biosciences PCR Array Data Analysis Web portal using the ΔΔCt method normalized to acidic ribosomal phosphoprotein P0 (RPLP0) expression. Heat map showing absolute expression of mRNA was generated using the SA Biosciences RT2 profiler PCR Array Data Analysis Web Portal.

### Statistical analysis

Statistical analyses were done using GraphPad Prism 5.0 software (La Jolla, CA). Colony morphology experiments, stain quantification, migration and invasion assays, and mRNA and protein levels were analyzed using ANOVA followed by Tukey’s post hoc test (for comparison between more than two groups) or Student’s *t*-test (for comparison between two groups). Proportions were analyzed via Fisher’s exact test. For all analyses, *p* < 0.05 was considered statistically significant.

## Results

### TBX3iso1 and TBX3iso2 are differentially expressed in the 21T cell lines

**Fig. 1:**
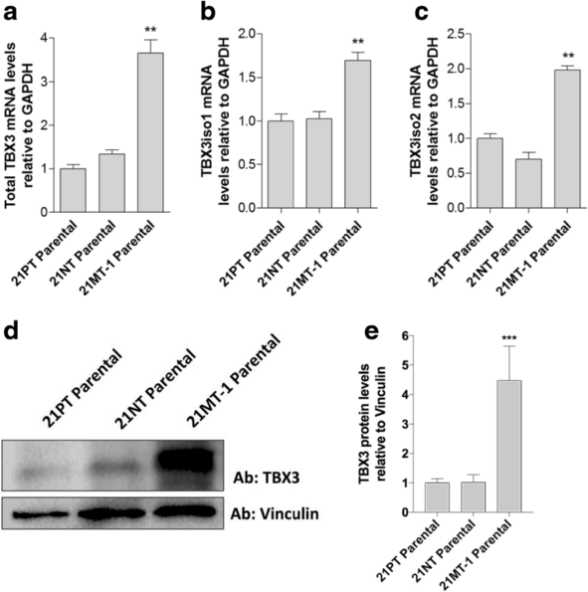
TBX3 isoform 1 and TBX3 isoform 2 are differentially expressed in the 21T cell lines. **a** Total TBX3 is differentially expressed in the 21T cell lines. Primers for total TBX3 mRNA were used to detect total TBX3 levels in the 21T cell lines. TBX3 is significantly higher in the 21MT-1 cells compared to the 21PT and 21NT cell lines and there is no significant difference in TBX3 expression between the 21PT and 21NT cells. **b** TBX3 isoform 1 is differentially expressed in the 21T cell lines. Primers specific for TBX3iso1 were used to detect TBX3iso1 levels in the 21T cell lines. TBX3iso1 is significantly higher in the 21MT-1 cells compared to the 21PT and 21NT cell lines and there is no significant difference in TBX3iso1 expression between the 21PT and 21NT cells. **c** TBX3 isoform 2 is differentially expressed in the 21T cell lines. Primers specific for TBX3iso2 were used to detect TBX3iso2 levels in the 21T cell lines. TBX3iso2 is significantly higher in the 21MT-1 cells compared to the 21PT and 21NT cell lines and there is no significant difference in TBX3iso2 expression between the 21PT and 21NT cells. **d**, **e** Total TBX3 protein is differentially expressed in the parental 21T cell lines. An antibody recognizing both isoforms of TBX3 was used to assess the relative abundance of TBX3 within the 21T cell lines. Total TBX3 protein expression is significantly higher in the 21MT-1 cells compared to the 21PT and 21NT cell lines and there is no significant difference in total TBX3 protein expression between the 21PT and 21NT cells. Means were analyzed using one-way ANOVA followed by Tukey’s post hoc test and *p* < 0.05 was considered statistically significant; ** *p* < 0.01. Error bars indicate Standard Error. Results are representative of at least three independent experiments

We have previously reported that TBX3 is present in the 21T cell lines and that its expression is increased in the 21MT-1 cell line representing IMC [7]. This finding is consistent with previous reports that TBX3 is upregulated in invasive stages of cancer and led us to examine TBX3 in detail using the 21T cell lines. Expression levels of TBX3 mRNA and TBX3 protein were examined in the parental 21T cell lines. Total TBX3 expression was found to be higher in the invasive 21MT-1 cells than in the non-invasive (21PT and 21NT) cell lines (Fig. 1a, d, e). Similarly, both TBX3iso1 and TBX3iso2 were found to be expressed at higher levels in 21MT-1 than in 21PT or 21NT cells (Fig. 1b, c). In contrast, no difference in either total TBX3 expression, or expression of either isoform, was seen between the ADH-like 21PT cells and DCIS-like 21NT cells (Fig. 1a-e).

### TBX3 expression is increased in 21NT (DCIS) cells after stable transfection

**Fig. 2:**
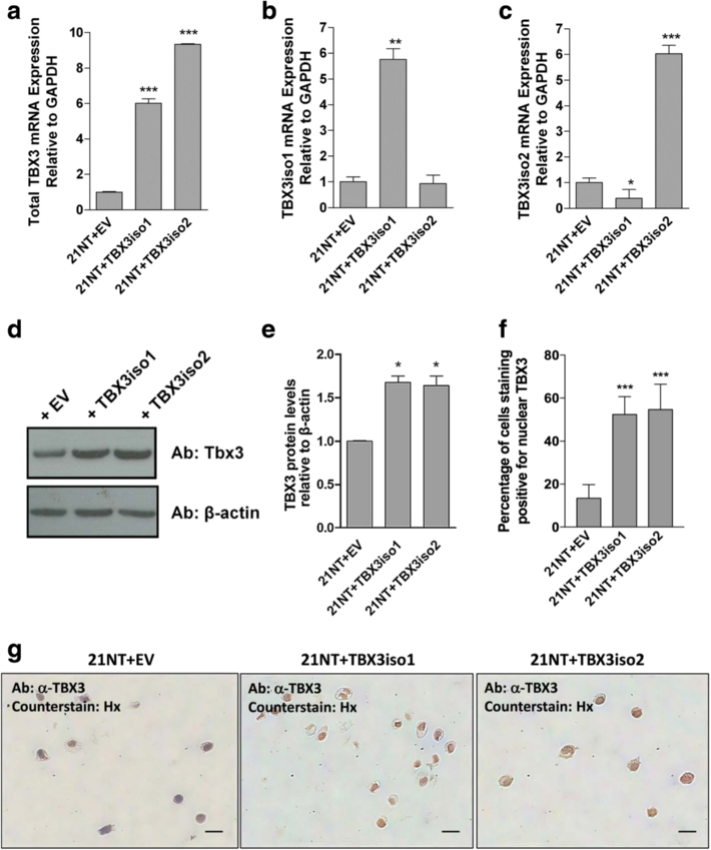
TBX3 expression is increased in 21NT cell transfectants. **a** qRT-PCR with primer sets which recognize both isoforms of TBX3 show total TBX3 mRNA is increased in 21NT cells after stable transfection with either TBX3iso1 or TBX3iso2. **b** qRT-PCR with TBX3iso1 specific primers show TBX3iso1 mRNA is increased in 21NT cells after stable transfection with TBX3iso1. **c** qRT-PCR with TBX3iso2 specific primers show TBX3iso2 mRNA is increased in 21NT cells after stable transfection with TBX3iso2. **d** Western blot showing total TBX3 protein is increased in the 21NT cells after stable transfection with either TBX3iso1 or TBX3iso2. **e** Densitometry quantification of Western blot shows significantly higher TBX3 protein expression in both the TBX3iso1 and TBX3iso2 transfectants. Means were analyzed using one-way ANOVA followed by Tukey’s post hoc test and *p* < 0.05 was considered statistically significant; * *p* < 0.05, ***p < 0.001. Error bars indicate Standard Error. Results of the qRT-PCR and Western blot are representative of at least three independent experiments. **f** Quantification of percentage of nuclei staining positive by immunohistochemistry for TBX3 in 21NT transfectant cell pellets. **g** Immunohistochemistry for TBX3 in 21NT transfectant cell pellets. TBX3iso1 and TBX3iso2 vs. vector control show increased amount of protein, which is mainly nuclear-localized

In order to test the effect of TBX3 on breast cancer progression, each TBX3 isoform was overexpressed in the 21NT cell line (representing DCIS). TBX3 expression vectors for each isoform were constructed and stably transfected into 21NT cells (21NT + TBX3iso1 and 21NT + TBX3iso2). An empty expression vector containing the same antibiotic resistant gene (neo) was also transfected into 21NT cells (21NT + EV). qRT-PCR was used to determine the level of each TBX3 isoform in transfected cells compared to empty vector controls. Total TBX3 mRNA was increased (at least 6-fold) in both 21NT + TBX3iso1 and 21NT + TBX3iso2 transfectants (Fig. 2a) and the overexpression of TBX3 was isoform specific in the transfectants (Fig. 2b, c). Likewise, Western blotting showed total TBX3 protein was increased in both the 21NT + TBX3iso1 cells and 21NT + TBX3iso2 cells, compared to the empty vector control (Fig. 2d, e). Cell pellets of TBX3 transfectants were assessed by immunohistochemistry and TBX3 protein was found to be concentrated in the nucleus, remarkably more so in the TBX3 transfectants, consistent with TBX3’s known function as a transcriptional regulator (Fig. 2f-g).

### TBX3 overexpression promotes progression of 21NT (DCIS) cells

**Fig. 3:**
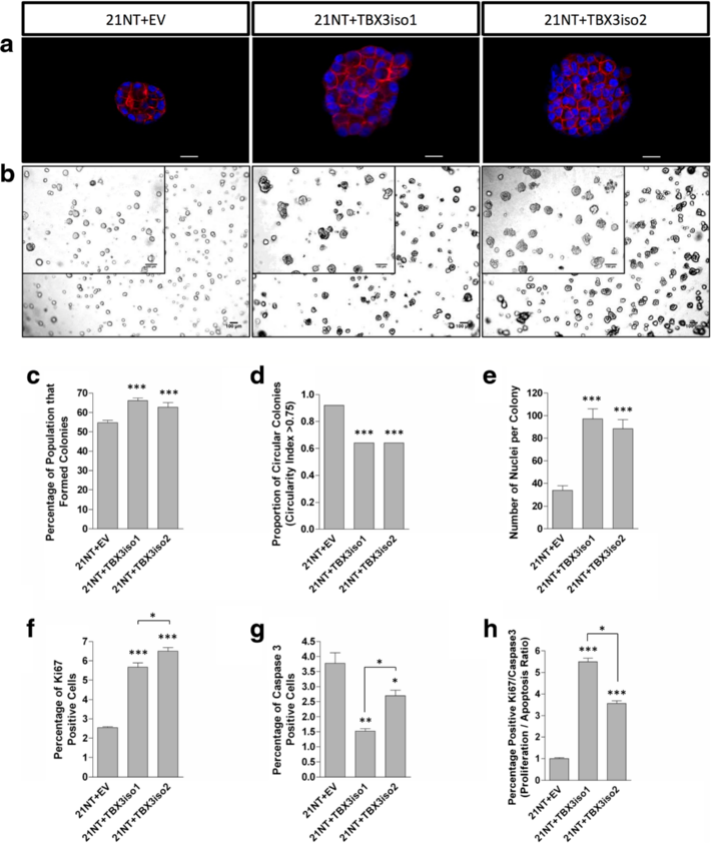
TBX3 overexpression in DCIS-like 21NT cells results in a more aggressive phenotype in 3D Matrigel. TBX3 transfectants were seeded in 3D Matrigel at 3000 cells per well and incubated at 37 °C and 5 % CO_2_ for 9 days. **a** Immunofluorescence images depicting colony size of 21NT transfectants. Cells were grown in Matrigel for 9 days, then stained with Hoechst and Phalloidin and imaged using 60X objective. Scale bars represent 20 μm. **b** Brightfield and phase contrast images showing growth of colonies after 9 days in 3D Matrigel. Brightfield images were taken using 4X objective, with the scale bar representing 100 μm. Phase contrast images (*inset*) were taken using 40X objective, with the scale bar representing 20 μm. **c** TBX3 overexpression increases colony formation rates. A colony was considered to be successfully formed when larger than 50 μm in diameter. Analysis was conducted using ImageJ. **d** TBX3 overexpression decreased the proportion of round colonies. Analysis was conducted using ImageJ. A binary quantification method was utilized; a circularity index above 0.75 was considered circular. **e** TBX3 overexpression increases the number of nuclei per cell colony. **f** TBX3 overexpression resulted in an increase in the percentage of Ki-67 positive cells within colonies. **g** TBX3 overexpression resulted in a decrease in the percentage of cleaved caspase 3 positive cells within colonies. **h** The proliferation/apoptosis ratio, defined as the percentage of positive Ki67 cells divided by the percentage of positive cleaved caspase three cells, increased with overexpression of both TBX3 isoforms. Means were analyzed using one-way ANOVA followed by Tukey’s post hoc test. The proportion of circular colonies was analyzed using Fisher’s exact test. A value of *p* < 0.05 was considered statistically significant; *p < 0.05, **p < 0.01, ***p < 0.001. Error bars indicate Standard Error. Results are representative of at least three independent experiments

To assess the role of TBX3 in the progression of DCIS to a more malignant phenotype, the 3D morphology of the 21NT transfectants was assessed using a 3D Matrigel assay. TBX3 overexpression greatly changed the phenotype of the colonies in 3D Matrigel (Fig. 3a, b). TBX3 overexpression increased the colony formation of the 21NT cells in a 3D Matrigel system from 55 % for the empty vector controls to 66 % for the 21NT + TBX3iso1 cells and 63 % for the 21NT + TBX3iso2 cells (Fig. 3c). As an index of the infiltrative vs. non-infiltrative nature of the colonies forming, we used a circularity index to determine the proportion of colonies that were round (non-infiltrative). We found that TBX3iso1 and TBX3iso2 overexpression decreased the proportion of round/circular colonies from 98 % for the empty vector controls to 64 % for the 21NT + TBX3iso1 cells and 64 % for the 21NT + TBX3iso2 cells (Fig. 3d) (i.e. less rounded, more infiltrative colonies). TBX3 overexpression also increased the number of nuclei per colony after 9 days from 34 nuclei per colony for the empty vector control to 97 nuclei per colony for the 21NT + TBX3iso1 cells and 89 nuclei per colony for the 21NT + TBX3iso2 cells (Fig. 3e). Examination of the colonies at day 9 by immunofluorescence for Ki67 and cleaved caspase 3 showed a significant increase in Ki67 (Fig. 3f) and decrease in cleaved caspase 3 (Fig. 3g) expression for both TBX3iso1 and TBX3iso2 transfectants vs. the negative control, resulting in an increase in the proliferation to apoptosis (Ki67/cleaved caspase 3) ratio for both TBX3 transfectants (Fig. 3h).

**Fig. 4:**
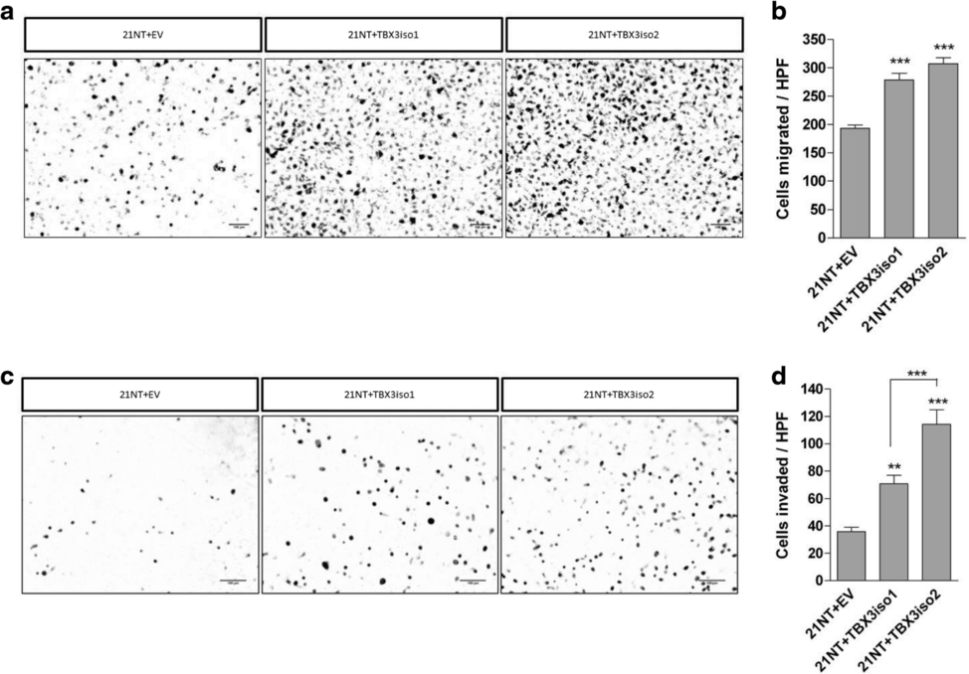
TBX3 overexpression increases migration and invasion of 21NT cells. **a**, **b** TBX3 overexpression increases migration using a transwell migration assay. 21NT cell transfectants were placed in the upper chamber of a transwell migration system with media containing 1 % BSA and allowed to migrate towards αHE10F chemoattractant in the bottom chamber for 22 h at 37 °C. Stable transfectants of both TBX3 isoforms showed an increase in migration using the transwell system. Scale bars represent 100 μm. Results are quantified in panel (**b**). **c**, **d** TBX3 overexpression increases invasion using a transwell invasion system. 21NT cell transfectants were placed in the upper chamber of a transwell invasion system with media containing 1 % BSA and allowed to invade towards αHE10F chemoattractant in the bottom chamber for 22 h at 37 °C. Stable transfectants of both TBX3 isoforms showed an increase in invasion using the transwell system. Scale bars represent 100 μm. Results are quantified in panel (**d**)

To further assess the migratory and invasive ability of TBX3-transfected 21NT cells, transwell assays were used. 21NT + EV, 21NT + TBX3iso1 and 21NT + TBX3iso2 cells were placed in the upper chamber of transwells (membranes with 8.0 μm pores pre-coated with either a thin layer of gelatin for migration assays, or Matrigel for invasion assays) and allowed to invade through the pores in order to reach 10 % FBS as an attractant in the lower chamber. TBX3 (iso1 and iso2) overexpression increased the 21NT cell’s ability to both migrate over gelatin (Fig. 4a, b) and invade through Matrigel (Fig. 4c, d).

### TBX3 knockdown reduces some characteristics of an aggressive phenotype in 21MT-1 (IMC) cells

**Fig. 5:**
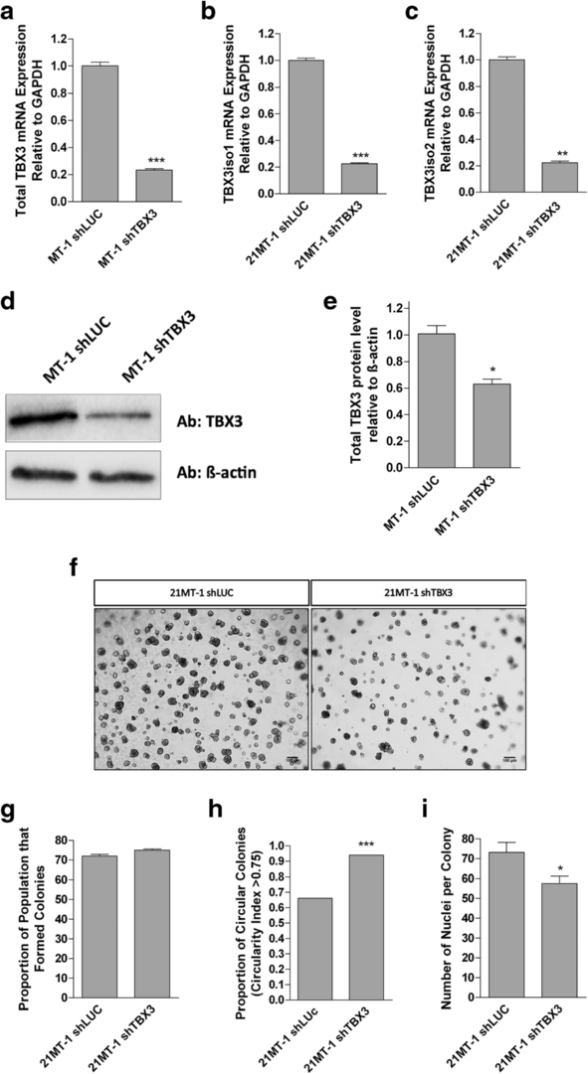
TBX3 knockdown results in a less aggressive phenotype of 21MT-1 (IMC) cells in 3D Matrigel. **a** Total TBX3 mRNA is decreased in 21MT-1 shTBX3 cells after TBX3 knockdown. **b** TBX3iso1 mRNA is significantly decreased in 21MT-1 shTBX3 after TBX3 knockdown. **c** TBX3iso2 mRNA is decreased in 21MT-1 after TBX3 knockdown. **d** Western blot showing total TBX3 protein is decreased in 21MT-1 shTBX3 cells after TBX3 knockdown. **e** Densitometry quantification of Western blot shows that there is a significant decrease in TBX3 protein expression in the 21MT-1 shTBX3 cells. **f** Brightfield images showing phenotype of colonies after 9 days in 3D Matrigel. Images were taken at 4X objective. Scale bars represent 100 μm. **g**, **h**, **i** Cells were seeded in Matrigel and grown for 9 days. All of the cell lines were able to form colonies (**g**), but shTBX3 colonies were rounder (i.e. less dispersed, less invasive) (**h**) and smaller (**i**). Means were analyzed using one-way ANOVA followed by Tukey’s post hoc test. The proportion of circular colonies was analyzed using Fisher’s exact test. A value of *p <* 0.05 was considered statistically significant; *p < 0.05, **p < 0.01, ***p < 0.001. Error bars indicate Standard Error. Results are representative of at least 3 independent experiments

To examine whether reduction of TBX3 could have an effect at a later stage of progression, TBX3 was knocked down in the 21MT-1 (IMC) cell line, using shRNA targeting both isoforms. TBX3 expression in the knockdown with the greatest reduction in both TBX3iso1 and TBX3iso2 mRNA expression is compared to the off-target (luciferase) control in Fig. 5a-c. qRT-PCR examination of mRNA from this knockdown showed a five-fold reduction in total TBX3 (Fig. 5a), TBX3iso1 (Fig. 5b), and TBX3iso2 (Fig. 5c). When examined at the level of protein expression by Western blotting (Fig. 5d, e), there was a corresponding reduction in Tbx3 protein. While the TBX3 knockdown cells were still able to form colonies at a rate comparable with the luciferase control-transfected cells in the 9-day Matrigel assay (Fig 5f, g), TBX3 knockdown resulted in an increased proportion of round/circular colonies (i.e. less dispersed/infiltrative; Fig. 5h) and fewer nuclei (cells) per colony (Fig. 5i), suggesting a reduction in invasiveness and impaired growth in 3D.

### Upregulation of TBX3 in 21NT (DCIS) cells results in alterations in expression of key regulatory and EMT/invasion associated genes

**Fig. 6:**
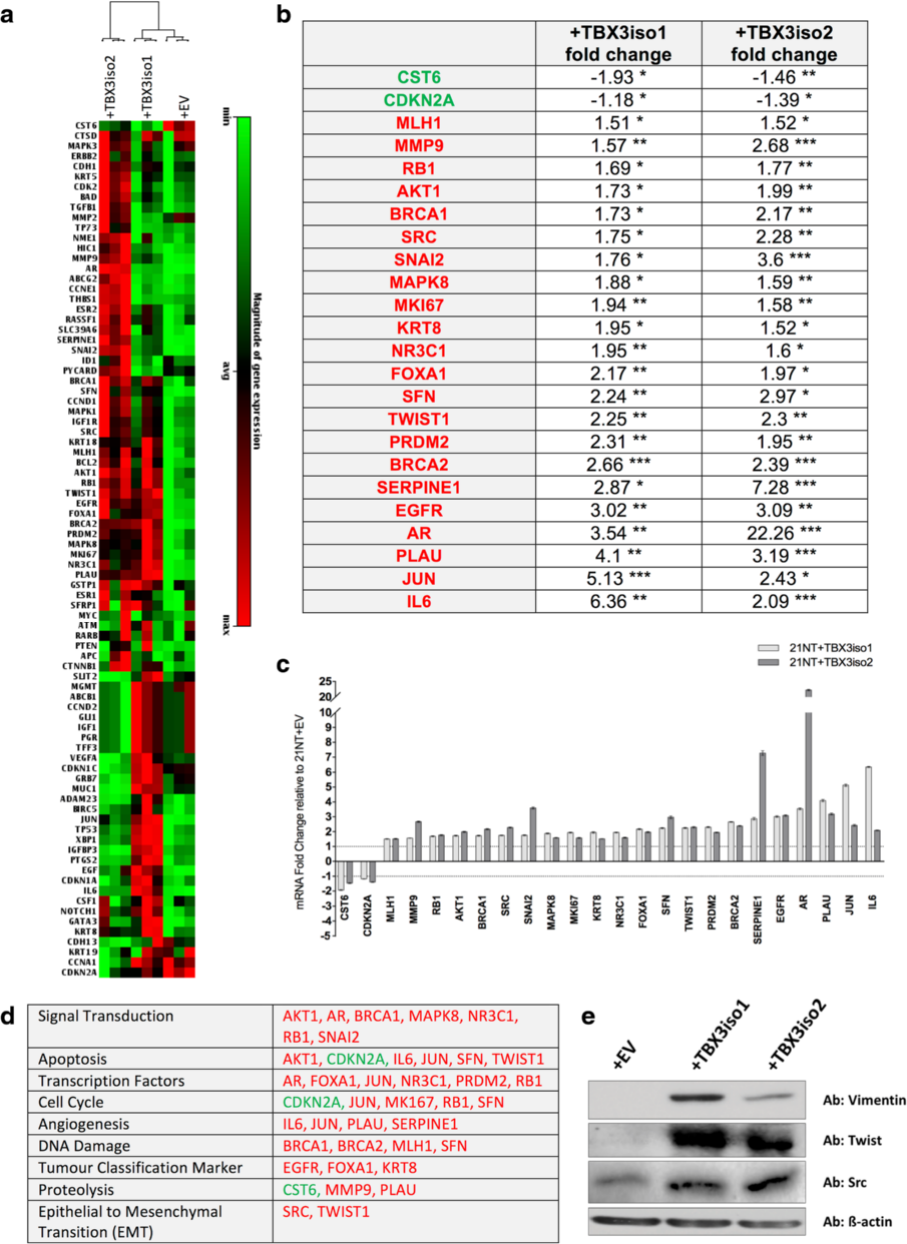
TBX3 overexpression in DCIS-like 21NT cells alters expression of key regulatory and EMT/invasion-associated genes. **a** Heat map (84 genes commonly dysregulated in breast cancer) showing absolute mRNA expression of 84 breast-cancer related genes within the 21NT transfectant cells. **b** Fold changes (mRNA) of genes significantly altered in expression in a similar fashion with overexpression of both TBX3iso1 and TBX3iso2 in 21NT cells. Data analysis was conducted by SA Biosciences PCR Array Data Analysis Web portal using the ΔΔCt method normalized to acidic ribosomal phosphoprotein P0 (RPLP0) expression. Fold changes are compared to 21NT + EV control and are organized based on increasing fold change of TBX3iso1. The 24 genes shown had similar alterations in gene expression with statistically significant fold changes for both isoforms (* *p* < 0.05, ** *p* < 0.01, ***p < 0.001). Genes in green had reduced expression, and genes in red had increased expression at the mRNA level with TBX3 isoform overexpression. Results are representative of 3 RT^2^ PCR arrays per cell line. **c** Graphical representation of fold changes in panel (**a**). *Dotted line* represents normalized expression of transcript levels of empty vector control cells (fold change = 1). **d** Gene expression changes with TBX3 isoform overexpression organized based on gene function groupings. Genes in green had reduced expression, and genes in red had increased expression at the mRNA level with TBX3 isoform overexpression. **e** Western blot showing increased expression of Vimentin, Twist and Src protein levels with TBX3 overexpression

We used RT^2^ PCR arrays to profile mRNA expression of TBX3iso1 and TBX3iso2 transfected 21NT cells, and found a number of alterations in expression of key regulatory and EMT/invasion-associated genes. As 21NT cells transfected with either TBX3iso1 or TBX3iso2 had shown a similar more aggressive phenotype, we focused our interest here on those genes whose expression changed in common in both TBX3iso1- and TBX3iso2-transfected cells. We identified altered expression of a number of genes involved in signal transduction, cell cycle control/cell survival and EMT/invasiveness (Fig. 6). In reference to pathways potentially involved in control of cell cycle, proliferation vs. apoptosis and cell survival, we observed significant down-regulation of CDKN2A (previously shown to be a key gene directly regulated by TBX3 in blocking cell senescence [12, 23]), with upregulation of marker of proliferation Ki67 (MKI67); Jun proto-oncogene (JUN); nuclear receptor subfamily 3, group C, member 1 glucocorticoid receptor (NR3C1); epidermal growth factor receptor (EGFR); androgen receptor (AR); interleukin-6 (IL-6); SRC proto-oncogene (SRC); and V-Akt murine thymoma viral oncogene homolog 1 (AKT1). Although the observed increase in retinoblastoma 1 (RB1), stratifin (SFN), breast cancer 1, early onset (BRCA1) and breast cancer 2, early onset (BRCA2) may seem counter-intuitive when the TBX3-upregulated cells are found to be actively cycling and faster-growing, it is likely that these are attempted adaptation responses to the more direct (according to previous literature) effect of CDKN2A down-regulation by TBX3, the expected result of which would be release of cell-cycle checkpoint control, inhibition of senescence and apoptosis and promotion of progression through the cell cycle and cell proliferation.

Several genes potentially associated with EMT and cellular invasiveness were also altered in transfectants of both isoforms. Transcriptional regulators twist family BHLH transcription factor 1 (TWIST1) and snail family zinc finger 2 (SNAI2) were both upregulated, as was SRC, and there was also altered expression of several proteases and protease inhibitors (including upregulation of plasminogen activator, urokinase (PLAU), serpin peptidase inhibitor, clade E, member 1 (SERPINE1), and matrix metallopeptidase 9 (MMP9); and downregulation of cystatin E/M (CST6)). Up-regulation of mesenchymal marker vimentin, as well as EMT-markers Twist and Src were confirmed at the protein level as well (Fig. 6e). Whether directly or indirectly, upregulation of either isoform of TBX3 in 21NT cells thus induced alterations in gene expression in pathways potentially involving cell cycle/cell survival/cell growth control and EMT/invasiveness, with the resulting in vitro phenotypes of better and larger colony formation, increased proliferation/apoptosis ratio and increased motility and invasiveness through Matrigel.

## Discussion

By using the 21T cell line series to model the effects of putative drivers of the transition from pre-invasive to invasive breast cancer progression, we are able to demonstrate that overexpression of TBX3 can promote the transition of DCIS to IMC. In particular, 21NT (DCIS-like) cells overexpressing TBX3 (either iso1 or iso2) showed increased colony-forming ability, with increased numbers of cells per colony and a more dispersed (less “rounded”) colony morphology. Increased cell invasiveness was also observed, both in terms of more dispersed colonies in 3D Matrigel and increased invasion through Matrigel in transwells. In parallel, down-regulation of TBX3 in shRNA transformants of 21MT-1 cells resulted in a less aggressive phenotype in 3D Matrigel, with smaller and less dispersed (less invasive) colony morphology.

No significant difference in functional activity in any of these in vitro measures of malignancy was observed between the two TBX3 isoforms. Interestingly, RT^2^ PCR array analysis did show several differences in gene expression profiles induced by iso1 vs. iso2, as well as a number of commonalities (Fig. 6a). As we saw no difference in functional effect, our analysis here was focused on gene expression alterations occurring in common between the two isoforms, which could potentially explain characteristics of the more aggressive phenotype seen in both TBX3iso1 and TBX3iso2 transfectants.

Firstly, the increased colony-forming ability in 3D Matrigel, with larger colony size and increased proliferation vs. apoptosis ratio seen after transfection of 21NT with both TBX3iso1 and TBX3iso2 indicated an increased cell survival and predisposition for proliferation vs. apoptosis (or senescence), effects that have been previously ascribed to TBX3 in other systems [10–12, 23]. Gene expression profiling of the TBX3 transfectants in our study showed altered expression of several genes potentially involved in these processes in both TBX3iso1 and TBX3iso2 transfected cells (i.e. downregulation of CDKN2A (p14^ARF^, p16^INK4A^), with upregulation of MKI67, JUN, NR3C1, EGFR, AR, IL-6, SRC and AKT1). Perhaps most interesting of these is the down regulation of CDKN2A (p14^ARF^, p16^INK4A^) by both TBX3 isoforms, as previous studies have indicated p14^ARF^ in particular to be a direct transcriptional target of TBX3 in other systems [23], and that isolated down-regulation of p14^ARF^ can accomplish the same effect of increasing cell proliferation, decreasing cell senescence and apoptosis [12]. Further work is required to determine whether p14^ARF^ or others of the candidate genes identified are responsible for the cell survival/proliferation vs. apoptosis/senescence aspects of the TBX3 phenotype in these cells.

Another prominent aspect of the altered phenotype seen with both TBX3iso1 and TBX3iso2 transfectants of 21NT cells was a more irregular/dispersed colony morphology in 3D Matrigel, with increased cellular invasiveness in transwell assays, suggestive of an EMT effect. In keeping with this, both TBX3iso1 and TBX3iso2 transfectants of 21NT cells showed alteration in expression of a number of EMT and invasion-related genes that would be consistent with an EMT phenomenon, including upregulation of TWIST1, SRC, SNAI2, PLAU, SERPINE1, and MMP9, with downregulation of CST6. Western blot analysis showed upregulation of Twist and Src, along with mesenchymal marker Vimentin at the protein level as well.

Our work is in agreement with that of previous studies with other mammary epithelial cells (MCF-7, MDA-MB-435, HC11) [24, 25] in which TBX3 was shown to have functional effects including inhibition of senescence and promotion of cell survival, proliferation and migration. We present the added novel finding, using a progression series of mammary epithelial cells, that overexpression of TBX3 can trigger non-invasive (DCIS-like, 21NT) cells to become invasive, possibly through an EMT-like process. In addition, knockdown of TBX3 in invasive (21MT-1) cells can revert them to a less aggressive phenotype, with less potential for 3D growth and a less infiltrative phenotype. Furthermore, we have shown that these effects are not isoform specific, as both TBX3iso1 and TBX3iso2 were able to induce the same phenotypes in transfected 21NT cells. Finally, examination of TBX3-induced changes in gene expression of 21NT cells has revealed specific cell survival/anti-senescence/proliferation and EMT/invasiveness category alterations, which may be involved in the functional changes observed. Future work will be necessary to establish which of these changes may be key players in the TBX3-induced phenotypes.

## Conclusions

We have here demonstrated a role for both isoforms of TBX3 (isoform 1 and 2) in promoting the transition from non-invasive to invasive breast cancer. We have shown that overexpression of TBX3 can alter cell properties involved in cell survival/colony formation and invasiveness, as well as regulate EMT/invasiveness-related genes. The identification of TBX3 as a potential regulator of early breast cancer progression from DCIS to IMC is of potential clinical utility not only in identifying which DCIS lesions may be more likely to progress to invasion, but along with a better understanding of the role of specific TBX3-induced gene expression changes, in providing other potential molecular targets to block breast cancer progression at this early, pre-invasive stage. The recent finding that TBX3 alterations may be key driver mutations in breast cancer, and the suggestion that altered (probably increased) TBX3 function may be associated with at least some cases of familial breast cancer [26], adds particular significance to the potential of TBX3 as an important regulator of breast cancer progression, and added urgency to a better understanding of its role in the malignancy of breast cancer.

## Abbreviations

αHE10F: αMEM with L-glutamine, insulin, epidermal growth factor, hydrocortisone, HEPES, sodium pyruvate, non-essential amino acid, gentamycin, and 10 % FBS
αMEM: Alpha modification of Eagle’s medium
ADH: Atypical ductal hyperplasia
AKT1: V-Akt murine thymoma viral oncogene homolog 1
AR: Androgen receptor
BRCA1: Breast cancer 1, early onset
BRCA2: Breast cancer 2, early onset
BSA: Bovine serum albumin
CDKN2A: Cyclin-dependent kinase inhibitor 2A, encodes p14^ARF^ and p16^INK4A^
CST6: Cystatin E/M
DCIS: Ductal carcinoma in situ
DOC: Deoxycholic acid
ECL: Enhanced chemiluminescence
EDTA: Ethylenediaminetetraacetic acid
EGFR: Epidermal growth factor receptor
EGTA: Ethylene glycol tetraacetic acid
EMT: Epithelial-mesenchymal transition
EV: Empty vector
FBS: Fetal bovine serum
FOXA1: Forkhead box protein A1
HEPES: 4-(2-hydroxyethyl)-1-piperazineethanesulfonic acid
IL-6: Interleukin-6
IMC: Invasive mammary carcinoma
JUN: Jun proto-oncogene
KRT8: Keratin 8, type II
MAPK8: Mitogen-activated protein kinase 8
MKI67: Marker of proliferation Ki67
MLH1: MutL homolog 1, colon cancer, nonpolyposis type 2
MMP9: Matrix metallopeptidase 9
NBF: Neutral buffered formalin
NR3C1: Nuclear receptor subfamily 3, group C, member 1 glucocorticoid receptor
P53: p53 tumor suppressor protein
PBS: Phosphate-buffered saline
PLAU: Plasminogen activator, urokinase
PRDM2: PR domain containing 2, with ZNF domain
RB1: Retinoblastoma 1
RIPA: Radioimmunoprecipitation assay
RPLP0: Acidic Ribosomal Phosphoprotein P0
SDS: Sodium dodecyl sulfate
SERPINE1: Serpin peptidase inhibitor, clade E, member 1
SFN: Stratifin
shRNA: Short hairpin RNA
SNAI2: Snail family zinc finger 2
SRC: SRC proto-oncogene
TBX3: T-box 3, with two isoforms: TBX3iso1 and TBX3iso2 (also known as TBX3 + 2a)
TWIST1: Twist family BHLH transcription factor 1
